# BlueDetect: An iBeacon-Enabled Scheme for Accurate and Energy-Efficient Indoor-Outdoor Detection and Seamless Location-Based Service

**DOI:** 10.3390/s16020268

**Published:** 2016-02-22

**Authors:** Han Zou, Hao Jiang, Yiwen Luo, Jianjie Zhu, Xiaoxuan Lu, Lihua Xie

**Affiliations:** 1 School of Electrical and Electronic Engineering, Nanyang Technological University, 50 Nanyang Ave, Singapore 639798, Singapore; zouh0005@ntu.edu.sg (H.Z.); yluo006@ntu.edu.sg (Y.L.); zhujianjie_1989@163.com (J.Z.); xlu010@ntu.edu.sg (X.L.); elhxie@ntu.edu.sg (L.X.); 2 College of Electrical Engineering and Automation, Fuzhou University, Fuzhou 350108, China

**Keywords:** indoor and outdoor detection, seamless location-based service, iBeacon

## Abstract

The location and contextual status (indoor or outdoor) is fundamental and critical information for upper-layer applications, such as activity recognition and location-based services (LBS) for individuals. In addition, optimizations of building management systems (BMS), such as the pre-cooling or heating process of the air-conditioning system according to the human traffic entering or exiting a building, can utilize the information, as well. The emerging mobile devices, which are equipped with various sensors, become a feasible and flexible platform to perform indoor-outdoor (IO) detection. However, power-hungry sensors, such as GPS and WiFi, should be used with caution due to the constrained battery storage on mobile device. We propose BlueDetect: an accurate, fast response and energy-efficient scheme for IO detection and seamless LBS running on the mobile device based on the emerging low-power iBeacon technology. By leveraging the on-broad Bluetooth module and our proposed algorithms, BlueDetect provides a precise IO detection service that can turn on/off on-board power-hungry sensors smartly and automatically, optimize their performances and reduce the power consumption of mobile devices simultaneously. Moreover, seamless positioning and navigation services can be realized by it, especially in a semi-outdoor environment, which cannot be achieved by GPS or an indoor positioning system (IPS) easily. We prototype BlueDetect on Android mobile devices and evaluate its performance comprehensively. The experimental results have validated the superiority of BlueDetect in terms of IO detection accuracy, localization accuracy and energy consumption.

## 1. Introduction

Nowadays, context-aware services and location-based services (LBS) provide essential information to entail a more convenient life. Additionally, this information, such as real-time occupancy distribution and human activity patterns, is also vital for “smart building”, which aims to reduce the energy consumption of buildings while optimizing occupants’ comfort [1,2]. By utilizing various on-board sensors, up-to-date mobile devices become crucial platforms to provide these services. In outdoor environments, GPS is widely used and delivers outstanding LBS for users [3]. When it comes to indoor environments, the past decade has witnessed flourishing research on indoor positioning systems (IPSs) [4,5]. A variety of sensing technologies have been proposed, including radio frequency identification (RFID) [6,7], WiFi [8,9,10,11,12], acoustic signals [13,14,15], geomagnetic fields [16,17], pedestrian dead reckoning (PDR) [18] and Bluetooth [19], which utilize on-board sensors, such as the accelerometer, gyroscope, microphone, WiFi module and Bluetooth module. WiFi is the primary alternative to GPS to perform indoor localization, since existing WiFi network infrastructures have been widely available in the majority of commercial and residential buildings, and more importantly, nearly every commercial mobile device is WiFi enabled. However, continuous WiFi scanning to perform indoor localization will draw a substantial amount of battery, which is not practical for long-term use. Luckily, iBeacon, as an emergency energy-efficient technology built upon Bluetooth Low Energy (BLE), could be a competitive alternative to WiFi to realize LBS in indoor environments.

GPS is widely used and delivers outstanding outdoor LBS. Extensive research and studies have been conducted in IPS, and some feasible solutions have been proposed. However, the majority of existing research adopts the common assumption that the operating environment is either indoor or outdoor and is known, which does not necessarily hold in reality. Some areas adjacent to buildings (covered corridor, connections between buildings) or semi-open buildings (parking garage) have partial characteristics of both indoor and outdoor environments. In this case, a sole dependence on GPS or IPS is unable to deliver a precise indoor-outdoor (IO) detection. In the meanwhile, an accurate and effective IO detection scheme provides basic, but critical information for upper-layer applications to serve individual users, e.g., mobile applications leveraging reliable IO status to give better services and alleviate battery consumption. For instance, the GPS module will be turned on only in outdoor and switched off in indoor by the IO detector to ensure its localization performance while avoiding large battery consumption. Similarly, the IO detector will only invoke WiFi Access Point (AP) scanning on the mobile device when the indoor environment is confirmed. Furthermore, an accurate and effective IO detection scheme could enable a seamless transition between outdoor and indoor LBSs. From the perspective of the building management, a precise IO detection scheme could measure the occupancy flow (going into or coming out of a building) to optimize the efficiency of building control systems, including the heating, ventilation and air-conditioning (HVAC) system and the lighting control system.

Nonetheless, the research towards IO detection itself is severely lacking. To our best knowledge, only two solutions have been proposed for IO detection [20,21]. One exploits the localization accuracy of GPS or the availability of GPS signals as an indicator to determine whether the device is in an indoor or outdoor environment [20]. The main drawback of the GPS-based IO detector (GPS-IO) is that the GPS module consumes much of the battery and is unreliable with a delayed response. The other solution, IODetector [21], is to employ various sensors equipped on mobile devices, including light, cellular, magnetic field, accelerometer and proximity sensors, to infer whether the context is indoors, outdoors or an intermediate zone. However, the hard-coded thresholds have been assigned for each sensor detector to infer the IO status, which does not always hold across different devices and in distinct environments.

In this paper, we propose BlueDetect as an accurate, fast response and energy-efficient scheme for IO detection and seamless LBS running on a mobile device based on the emerging low-power iBeacon technology. iBeacon is an advanced Bluetooth protocol proposed by Apple [22], which is built upon Bluetooth Low Energy (BLE). In our system, only a few small-sized, low-cost and battery-powered BLE beacons are required by BlueDetect, which are placed at landmarks, such as the boundary of covered corridors and entrances/exits of buildings, with a sparse density in intermediate regions between indoor and outdoor environments (classified as a semi-outdoor environment). The GPS module is turned on for LBS only in the outdoor environment. When it comes from outdoors to semi-outdoors, the decrease of the mean GPS signals is utilized as a trigger to turn off GPS and turn on Bluetooth, and the iBeacon mode of BlueDetect is responsible for providing LBS within semi-outdoor environments. Transitions between semi-outdoor and indoor environments are achieved seamlessly by comparing the signals of two BLE beacons placed on both sides of the entrance of the building.

We implement and evaluate BlueDetect on the Android platform using different mobile devices. We test it in a university campus where various environment scenes are included. The experimental results show that BlueDetect provides a higher context detection accuracy than GPS-IO and IODetector. Furthermore, it also delivers accurate and reliable positioning service in semi-outdoor environments. The power consumption of BlueDetect is evaluated as well. Owing to the energy-efficient property of iBeacon, BlueDetect consumes the minimum power among the three approaches. A video demo is presented to demonstrate the seamless navigation service using BlueDetect [23].

The rest of the paper is organized as follows. We provide an overview of related works in Section 2. In Section 3, the motivation and preliminary of this work is described. We present the system design of BlueDetect in Section 4. A comprehensive evaluation of BlueDetect in terms of IO detection accuracy, localization accuracy and power consumption is reported in Section 5. We conclude this paper in Section 6.

## 2. Related Work

In this section, we first present an overview of existing IO detection schemes; one leverages the availability of GPS signals as the indicator to determine the status of indoors or outdoors; another employs various sensors equipped on mobile devices to infer the IO status. Furthermore, we present a summary of various technologies for location-based services.

### 2.1. GPS-Based IO Detection

Generally, GPS supplies reliable and outstanding localization and navigation services for users in outdoor environments when more than four visible satellites are available. On the contrary, since the line-of-sight paths between the mobile device and satellites are usually blocked in indoor environments, the performance of GPS is jeopardized. According to these facts, the localization accuracy of GPS or the availability of GPS signals is leveraged as an indicator to determine the status of indoors or outdoors [9,20,24,25].

The GPS-based IO detection suffers from several drawbacks. First of all, its classification accuracy is not reliable, especially in some indoor environments with large windows. The reason is that the number of visible satellites in this area is greater than that in other indoor environments; therefore, the status of users will be misclassified into the outdoor environment if the availability of GPS signals is the sole indicator for IO detection. Secondly, the bootstrap process of the GPS module takes around one minute in general. Its performance is largely deteriorated during this slow process. Furthermore, GPS-based IO detection is not an energy-efficient method. The GPS module is the most power-hungry sensor in a mobile device, which is not worth being turned on as an IO hint all of the time, from the energy saving perspective. In particular, GPS will consume much power in indoor environments, since it requires conducting GPS satellite scanning continuously until a sufficient number of satellites have been connected to perform localization.

In summary, GPS-based IO detection is venerable, ineffective, energy consuming and impractical.

### 2.2. Lightweight On-Board Sensor-Based IO Detection

Another category is to leverage various sensors equipped on the mobile device to determine whether the environment status is indoors, outdoors or intermediate zone [21,26]. IODetector [21] mainly utilizes three on-board sensors, light, cell and magnetic field sensors, to detect the IO status. According to their observations, high light intensity indicates outdoor environments, and low light intensity represents indoor environments in general; the fluctuation of magnetic field intensity is higher in indoor than outdoor environments due to severe disturbances from steel structures and the chaotic electromagnetic fields generated by electric devices in indoor environments; the cell RSS of the user’s mobile device drops rapidly from outdoor to indoor environments because of the attenuation of walls and ceilings. These three detectors provide their individual estimates and corresponding confidence in those estimates. Then, IODetector aggregates these results, and the final estimation is the status with the best overall confidence in estimations. A first-order hidden Markov model is also employed to realize a stateful IODetector in [21], although only a slight enhancement was achieved compared to the stateless one. The main drawback of IODetector is that it leverages hard thresholds for each sensor feature to determine indoor or outdoor environments. The accuracy of the estimations across different devices and environments is negatively impacted.

Besides the three sensors employed in IODetector [21], other sensor features, including sound intensity from the microphone, temperature from the battery thermometer and the proximity sensor are utilized for IO detection in [26]. Furthermore, co-training, as a semi-supervised learning method, was adopted to improve the IO estimation accuracy across different devices and environments.

### 2.3. Technologies for Location-Based Services

Various technologies have been exploited for LBS. The main characteristics of these technologies, including localization accuracy, suitable environment, cost, power consumption and the requirement of extra devices on the user-side, are summarized in Table 1. The location estimation can be further classified into outdoor and indoor localization. Global navigation satellite systems (GNSS) [3], such as GPS, BeiDou and GLONASS, is able to provide reliable outdoor LBS for users. Nevertheless, continuous scanning of satellites consumes a huge amount of battery of the mobile devices. Some research aims to use GSM as a complement of GNSS to improve the localization accuracy and reduce the power consumption for outdoor LBS [27]. In the past two decades, great efforts have been devoted to developing IPSs [4,28,29]. A variety of sensing technologies have been exploited and leveraged to provide indoor LBS. The work in [30] employs infrared to realize indoor localization. Although it is able to achieve around a 1-m localization accuracy, the high cost of the time-of-flight measurement infrastructure makes it inaccessible for large-scale commercial applications. IPSs adopting ultrasound, such as Cricket [31] and Active Bat [32], have been proposed, as well. The work in [15] leverages high-pitched acoustic signals emitted from commercial mobile devices to realize indoor tracking. For acoustic-based IPS, receivers usually are required to be placed at the ceiling or on walls to detect the signal. RFID [6,7] and ultra-wideband (UWB) [33] IPSs utilize RF signals to perform indoor localization. The localization accuracy of these systems can achieve around 1 m. Nevertheless, all of them require user to carry an extra device (a tag or a receiver) to realize indoor positioning. With the widespread network infrastructures in most of the commercial and residential buildings, as well as the availability of the WiFi module on nearly every commercial mobile device, WiFi has been adopted in many IPSs as a primary alternative to GPS for indoor LBS [8,9,10,11,12]. PDR [18] utilizes other on-board sensors, including the accelerometer, gyroscope and magnetometer, to realize indoor tracking. However, continuous WiFi scanning and inertial measurement unit (IMU) sensing still draw a substantial amount of battery, which is not practical for long-term use.

Fortunately, iBeacon, as an emergency energy-efficient technology built upon BLE, could be a competitive alternative to realize LBS in indoor environments. Similar to WiFi, the Bluetooth module is available on most commercial mobile devices. Therefore, no extra device is required on the user side. Furthermore, since the beacons broadcast their unique identifiers to nearby portable mobile devices and trigger a location-based action on these devices, no paired connection with the mobile device is needed [22]. This low-energy data communication protocol is much more power efficient than classical Bluetooth protocols. Thus, it is a competitive technology in terms of an acceptable localization accuracy and less power consumption than GPS and WiFi on the user side [34].

## 3. Motivation and Preliminary

Precise context status is fundamental for upper-layer applications. When users are coming from indoors to outdoors, an IO detection scheme can turn on GPS and turn off WiFi AP searching smartly to save power after it confirms the outdoor status. Similarly, IPSs will only be bootstrapped until the IO detector confirms that it is in an indoor environment for energy saving. The performance of other applications, including context recognition, human activity recognition, seamless navigation between outdoors and indoors and geo-fencing, are also dependent on how accurate the IO detection scheme is.

**Figure 1:**
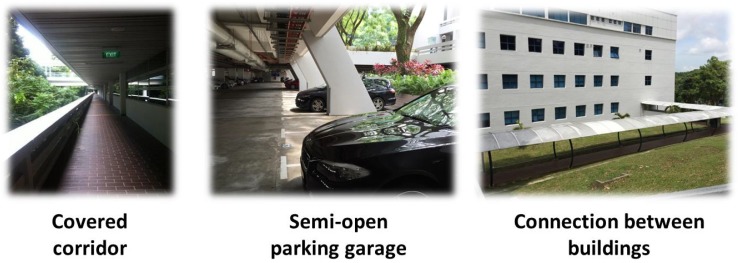
Typical scenes of semi-outdoor environments.

Before introducing our system design, we first classify the environment types in this work. Similar to [21], in addition to the outdoor state (outside a building) and indoor state (inside a building), we define one more environment type, the semi-outdoor status, which is the case when a client is adjacent to a building or in a semi-open building. Typical semi-outdoor environments include covered corridors, connections between buildings, semi-open lobbies and semi-open parking garages, as shown in Figure 1.

A semi-outdoor environment assumes certain characteristics of both indoor and outdoor environments, and its definition can be justified as follows. A mobile device can still receive weak GPS signals from a few satellites in covered corridors, but the performance of GPS in this area is severely compromised compared to a pure outdoor environment due to the decreased number of visible satellites and the multi-path effect. On the other hand, although the mobile device may detect a few WiFi APs with poor connection in semi-outdoor environments, such as semi-open parking garages and connections between buildings, the performance of WiFi-based IPS is unsatisfactory due to the low density and unreliable connection with these APs in the area. Hence, both GPS and WiFi modules should be switched off in semi-outdoor areas for the purpose of energy saving.

## 4. System Design

In this section, we present an overview of the BlueDetect system firstly. After that, we introduce the design details of transition methodologies between outdoors/semi-outdoors/indoors and the iBeacon mode of BlueDetect for semi-outdoor environments, respectively.

### 4.1. System Overview

**Figure 2:**
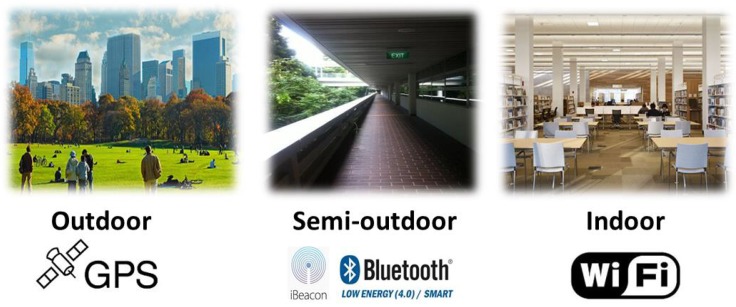
Representative scenes and corresponding localization technologies of three different environments.

Figure 2 illustrates the representative scenes of outdoor, semi-outdoor and indoor environments, as well as the localization techniques employed by BlueDetect in these three environments. For pure outdoor environments, only the GPS module will be turned on when users query their location information. When it comes from outdoor to semi-outdoor, the decrease of mean GPS signals is utilized as a trigger to turn on Bluetooth. Once the semi-outdoor state is confirmed, GPS is turned off for energy saving, and then, the iBeacon mode of BlueDetect will provide localization service in semi-outdoor environments. Only a few of the BLE beacons are required to be deployed to facilitate context detection and LBS in this area. The detailed localization algorithm is presented in Section 4.4. In the transition from semi-outdoors to outdoors, the signals of BLE beacons deployed at the boundary of semi-outdoor environments will be detected. GPS will be turned on, and Bluetooth will be switched off for energy saving when no beacon signal can be detected by users’ mobile devices. The transitions between semi-outdoor and indoor are achieved seamlessly and easily by deploying two BLE beacons at two sides of each entrance of indoor environments as landmarks. Whether the user is going into or out from an indoor environment can be inferred by comparing the RSSs of the two beacons. Since WiFi-based IPS [11,35] is the most practical system for indoor localization, which reuses existing WiFi infrastructures and can be applied directly for most mobile devices, we leverage it to provide indoor LBS when the indoor status is confirmed. The detailed transition methodologies between outdoors and semi-outdoors, semi-outdoors and indoors are introduced in Section 4.2 and Section 4.3, respectively. The localization algorithm of BlueDetect in the semi-outdoor environment is described elaborately in Section 4.4.

### 4.2. Seamless Transition between Outdoors and Semi-Outdoors

For pure outdoor environments, GPS is capable of providing sufficient positioning accuracy. When it comes to semi-outdoor or indoor environments, the number of visible satellites is decreased, and a significant decrease of the GPS signals is expected due to the block of the line-of-sight between the satellites and mobile device. The performance of GPS is jeopardized dramatically while draining the battery at a high power rate under this circumstance. Therefore, BlueDetect will turn off the GPS module in both semi-outdoor and indoor environments.

**Figure 3:**
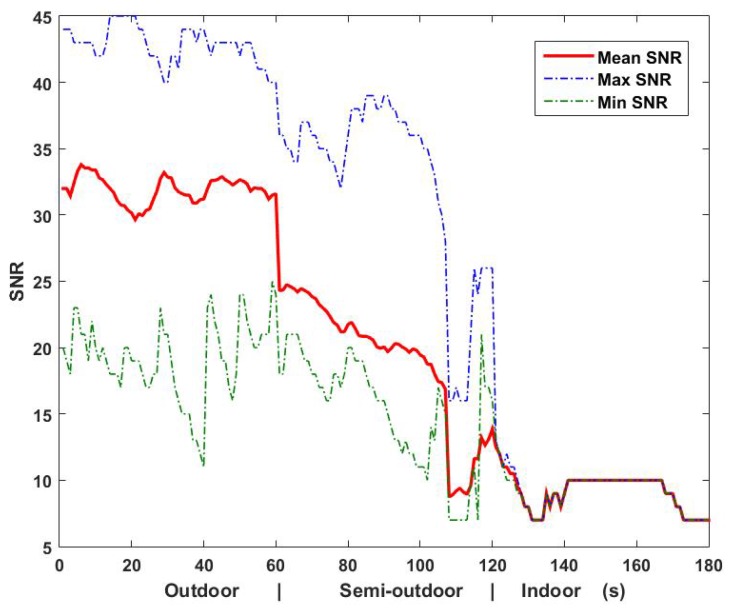
SNR of GPS signals in different environments.

We conducted experiments to analyze the variation of GPS signals when a client with a mobile device was moving from outdoor to indoor environments (three experiments were conducted in a covered corridor, a connection between buildings and a semi-open parking garage). Figure 3 illustrates the maximum, mean and minimum of GPS SNR readings from visible satellites in outdoor, semi-outdoor and indoor environments. As shown in Figure 3, the value of mean SNR dropped more than 20% when coming from the outdoor to the semi-outdoor environment. Clearly, it is more suitable than the other two values to be used as a trigger indicating the switching of environments. Algorithm 1 shows the transition methodology between outdoors and semi-outdoors of BlueDetect.

**Algorithm 1** BlueDetect IO detection and localization algorithm (outdoors ⇌ semi-outdoors).
**input**: *B* - Bluetooth signal (iBeacon), *G* - GPS signal, $\sigma_{s}$ - switching threshold, $\sigma_{t}$ - duration threshold**output**: Location of the mobile device**case** *Outdoor ⇒ Semi-outdoor*    **if** $G_{current}<\sigma_{s}*G_{previous}$ for $\sigma_{t}$ **then**        Turn on Bluetooth;        **if** No less than 2 beacons’ $B>B_{min}$ **then**           Turn off GPS;           Utilize *B* for localization;           *Environment Type* ← *Semi-outdoor*        **else**           Turn off Bluetooth;           Utilize *G* for localization;           *Environment Type* ← *Outdoor*        **end** **if**    **end** **if****case** *Semi-outdoor ⇒ Outdoor*    **if** $B\in B_{boundary}$ **then**        Turn off Bluetooth, Turn on GPS;        Utilize *G* for localization;        *Environment Type* ← *Outdoor*    **else**        Utilize *B* for localization;        *Environment Type* ← *Semi-outdoor*    **end** **if**


If the decline of mean GPS SNRs is detected and the value of the decrease is larger than the switching threshold $\sigma_{s}=80\%$ for three consecutive samples (sampling rate: 645 ms/sample), the Bluetooth module will be turned on searching for BLE beacons. When no less than two beacons are detected and their RSSs are larger than $B_{min}$ = −70 dBm (the detailed methodology of setting $B_{min}$ is presented in Section 4.4), it is confirmed that the mobile device is in the semi-outdoor environment, followed by turning off the GPS to save energy and activating the iBeacon mode of BlueDetect for positioning. Otherwise, Bluetooth will be turned off, and GPS will still be used for localization.

Conversely, when the RSSs of BLE beacons installed at the boundary of the semi-outdoor environment are detected ($B\in B_{boundary}$), the intention of going outdoors can be inferred, and then, the GPS module will be turned on for outdoor LBS in advance for a seamless transition. If no BLE beacon signal is detected for a period of longer than a threshold $\sigma_{t}$, which is four consecutive samples (sampling rate: 645 ms/sample) and a relatively stable GPS signal is maintained, the Bluetooth will be switched off.

### 4.3. Seamless Transition between Semi-Outdoors and Indoors

Algorithm 2 shows the transition methodologies between semi-outdoors and indoors of BlueDetect. In order to achieve seamless transition between semi-outdoors and indoors, one pair of BLE beacons is deployed at two sides of each door. For each pair, the one placed outside the door is employed as a landmark of the entrances/exits, and the other one is installed 3–5 m away from the door inside the indoor environment. When the client’s mobile device detects the signals of these two beacons, the intention of going into or out of indoor environments can be determined by comparing the RSSs of the two beacons. To be specific, if $B_{indoor}>B_{semi-outdoor}$ maintains for $\sigma_{t}$, which is four consecutive samples (sampling rate: 645 ms/sample), it indicates that the user is entering into an indoor environment with high confidence. Therefore, BlueDetect will turn on the WiFi module of the mobile device to perform WiFi AP scanning for an Internet connection through the WiFi network and leverage WiFi-based IPS for indoor positioning. Conversely, when $B_{indoor}<B_{semi-outdoor}$ for $\sigma_{t}$, we confirm that the client is going out of the indoor to the semi-outdoor environment, and the WiFi module will be turned off, which the iBeacon mode of BlueDetect is activated for localization.

**Algorithm 2** BlueDetect IO detection and localization algorithm (Semi-outdoors ⇌ indoors).
**input**: *B* - Bluetooth signal (iBeacon), *W* - WiFi signal, $\sigma_{t}$ - duration threshold**output**: Location of the mobile device**if** $B_{indoor}<B_{semi-outdoor}$ for $\sigma_{t}$ **then**    Turn off WiFi;    Utilize *B* for localization;    *Environment Type* ← *Semi-outdoor***else**    Utilize *W* for localization;    *Environment Type* ← *Indoor***end** **if**


### 4.4. Semi-Outdoors (iBeacon)

Reliable LBS in a semi-outdoor environment is not readily available since neither GPS nor IPS can perform satisfactorily in this scenario. We employ the emerging iBeacon technology to fill in this gap. iBeacons make use of BLE proximity sensing to broadcast their unique identifiers to nearby portable mobile devices and trigger a location-based action on these devices. Since the iBeacon protocol uses very short duration messages and does not need a paired connection with mobile devices (broadcast only), it is much more power efficient than classical Bluetooth protocols and less power hungry on the user-side than GPS and WiFi [34]. With such a merit, a BLE beacon can run on a coin cell battery for months or even for years. According to a recent study on the battery life of 16 major iBeacon hardware devices [36], by setting the advertising interval as 645 ms, an iBeacon with a CR2450 620-mAh coin cell battery is able to provide 11.2 months of life, which increases to two years as the advertising interval is increased to 900 ms. Nowadays, the iBeacon protocol is becoming a built-in standard for mobile devices, and a high density deployment of BLE beacons in buildings for multiple purposes will be expected in the near future.

For the iBeacon mode of BlueDetect, only a few of the portable, low-cost and battery-powered BLE beacons are deployed as landmarks in semi-outdoor environments for context detection, as well as localization and navigation. From the energy saving perspective, both GPS and WiFi modules of mobile devices are turned off when the semi-outdoor status is confirmed, since they supply no valuable information for IO detection and LBS under this circumstance. The common geographical structure of a semi-outdoor environment, such as a corridor or a sky bridge, is elongated; thus, a sparse deployment of BLE beacons is adequate to cover the entire area. According to the iBeacon protocol, the unique identifying information of each beacon is proximity universally unique identifier (UUID), major value and minor value. These parameters can be used to identify the building, the floor and the exact location of each BLE beacon. With the RSSs and locations of these beacons, we leverage weighted path loss (WPL) [7], a log-distance path loss model-based localization algorithm, to estimate the real-time location of a client’s mobile device in semi-outdoor environments. The methodology of WPL is described as follows: suppose a client’s mobile device receives RSS from *n* BLE beacons simultaneously. The RSS of *i*-th iBeacon $B_{i}$ can be expressed as:

(1) $$B_{i}=P_{0}-10\alpha log\left(d_{i}\right)+X_{\sigma },1\leq i\leq n$$

where the reference path loss coefficient $P_{0}$ and the path loss exponent *α* need to be calibrated, and $X_{\sigma }$ represents a zero Gaussian random noise with standard deviation *σ*. Then, based on Equation (1), the distance $d_{i}$ between the mobile device and the *i*-th iBeacon is calculated by:

(2) $$d_{i}=10^{\frac{P_{0}-B_{i}+X_{\sigma }}{10\alpha }}$$

The real-time estimated location of the mobile device, (x,y), is computed by:

(3) $$\left(x,y\right)=\frac{1}{c}\sum_{i=1}^{n}\frac{1}{d_{i}}\left(x_{i},y_{i}\right)$$

where $c=\sum_{i=1}^{n}\frac{1}{d_{i}}$ is the normalization constant and $\left(x_{i},y_{i}\right)$ indicates the physical coordinates of the *i*-th iBeacon.

In an effort to make the log-distance path loss model robust in a semi-outdoor environment, we first conducted experiments to analyze the effects of Non-line-of-sight (NLOS) and the orientation of mobile device on the RSS from BLE beacons.

We performed an experiment in a covered corridor (typical semi-outdoor environment) to analyze the effects of NLOS firstly. The signal strengths were measured at several different distances away from a beacon, which was attached at the ceiling of the corridor. At each location, one user carried a mobile device (Nexus 6) facing toward the beacon, and 100 RSS samples were collected under the line-of-sight (LOS) condition. In addition, another 100 RSS samples was collected when another occupant was standing between the user and the beacon to block the LOS as an NLOS condition. Table 2 compares the mean RSS values under both LOS and NLOS conditions at 1–9 meters away from the beacon. As shown in Table 2, the NLOS RSS value is 4–5 dBm smaller than the LOS RSS value at each location, because the obstacle (occupant) attenuated the signal strengths. Therefore, we further consider the NLOS effects in the process of log-distance path loss modeling for localization in a semi-outdoor environment.

In addition, we conducted an experiment in the covered corridor to evaluate the influence of different orientations of mobile devices on the RSS emitted from Beacons. Similar to the last experiment, we recorded the signal strengths at several different distances away from a beacon placed at the ceiling of corridor. At each location, one user carried a mobile device (Nexus 6) facing four different orientations ($0^{\circ }$, $90^{\circ }$, $180^{\circ }$, $270^{\circ }$) to measure the RSS values. 100 RSS samples were recorded at each orientation. Table 3 compares the mean RSS values of four orientations at nine distinct locations from a beacon. It can be observed from Table 3 that the RSS values of the $0^{\circ }$ holding orientation at all locations are largest among the four directions, because the LOS condition is satisfied. On the other hand, the RSS values of $180^{\circ }$ are smallest, since the user blocked the LOS. The RSS values recorded at the orientations of $90^{\circ }$ and $270^{\circ }$ are usually similar, because their signal transmission conditions are similar. In summary, the average RSS variation caused by different holding orientations of the mobile device is 1.929 dBm, which should not be ignored for log-distance path loss modeling.

**Figure 4:**
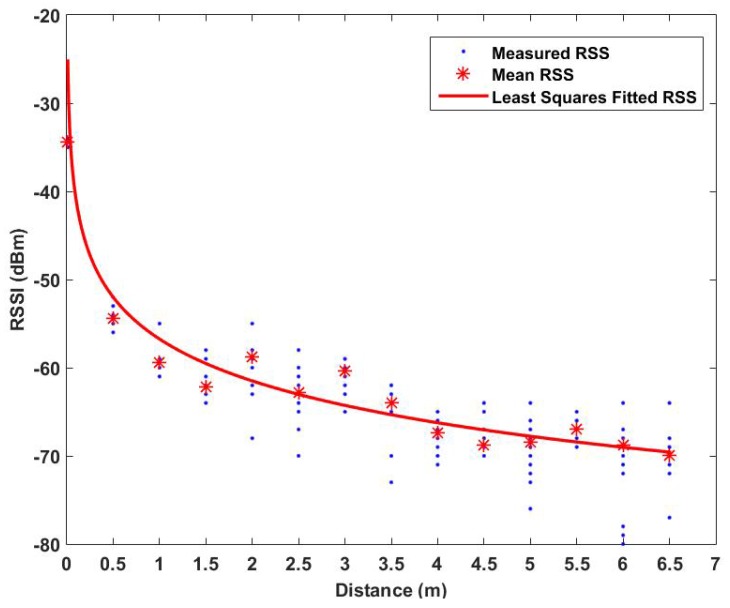
Relationship between RSS of a BLE beacon and distance.

Therefore, we include the effects of NLOS and different holding orientations of the mobile device to precisely estimate the parameters $P_{0}$ and *α* in the log-distance path loss model for semi-outdoor localization. To be specific, we measured RSSs at 14 different distances away from a BLE beacon put at the ceiling of the corridor. At each location, one user carried a mobile device facing four different orientations with distinct LOS conditions, ($0^{\circ }$, total LOS; $180^{\circ }$, total NLOS; $90^{\circ }$ and $270^{\circ }$, partial LOS), to measure the RSS values. 100 RSS samples were recorded at each orientation. $P_{0}$ is determined as the mean RSS value at a 0.5-m distance, and *α* is estimated by the least-squares method. Based on our experimental results, the estimated values of $P_{0}$ is −56.75 dBm and *α* is 1.577. The raw RSS measurements and mean RSS values at each reference point are demonstrated in Figure 4, with the curve fitting by the least-squares method. As shown in Figure 4, the RSS value decreases to −70 dBm and remains at this level after 6.5 m. Therefore, we define the minimum effective RSS broadcast from each iBeacon to be $B_{min}$ = −70 dBm. Accordingly, BLE beacons only need to be placed with 7–10-m intervals for localization in a semi-outdoor environment.

## 5. Evaluation

**Figure 5:**
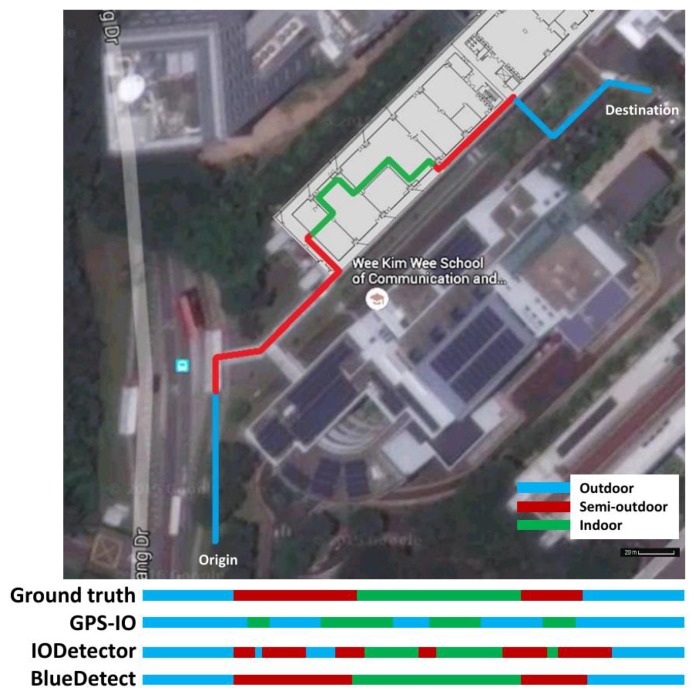
The test walking route in the university campus and indoor-outdoor (IO) detection accuracy comparison.

**Figure 6:**
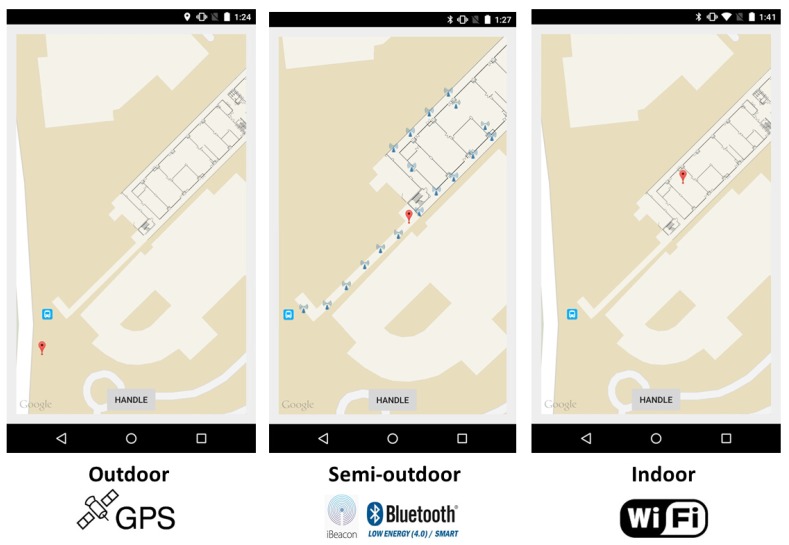
Screenshots of BlueDetect in the three environment types.

We implemented a prototype of BlueDetect on the Android platform and tested its performance using two different mobile devices (Nexus 6 and Samsung Galaxy S4). These devices support the BLE protocol and operate with Android 5.0 OS. The experiments were conducted in the campus of Nanyang Technological University. As shown in Figure 5, the test walking route includes all three types of environment scenes: indoors (green section), semi-outdoors (red section), outdoors (blue section), with the total route length around 450 m. The test walking route was specifically designed to include all transition scenarios. Figure 6 demonstrates the screenshots of BlueDetect in different environment scenes and the corresponding localization techniques adopted.

**Figure 7:**
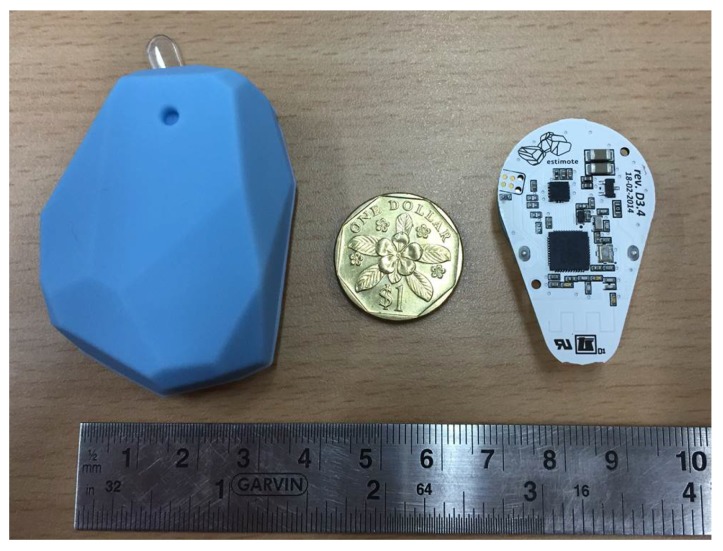
Estimote BLE Beacon.

In the semi-outdoor environment, only 12 low-cost and power-efficient Estimote BLE beacons [37] were placed to cover the total 350 m${}^{2}$ semi-outdoor area (including covered corridors and connections between buildings). The Estimote BLE beacon is equipped with Nordic Semiconductor’s NF51822 chipset, by setting these beacons to a transmission power of −12 dBmW and an advertising interval of 645 ms [37]. Each beacon can provide 21.4 months of life with a CR2477 1000 mAh coin cell battery. As shown in Figure 7, the small-sized and battery-powered Estimote BLE beacons can be placed at the ceiling of corridors or attached to walls without cumbersome deployment process. As mentioned in Section 4.3, two pairs of beacons were installed at both sides of the two entrances for detecting seamless transition between semi-outdoor and indoor environments. Our WiFi-based IPS [11,35] is adopted to provide indoor LBS when the indoor environment is confirmed by BlueDetect. During the experiments, the user was walking along the route 10 times at different periods of the day under different weather conditions for a comprehensive evaluation of our system. The following sections will present the performance evaluation of BlueDetect in terms of IO detection accuracy, localization accuracy in a semi-outdoor environment and power consumption, respectively.

### 5.1. IO Detection Accuracy of BlueDetect

To validate the contextual detection accuracy of BlueDetect, we used a camera to record the entire walking process. In this way, we obtained the real contextual information (outdoors, semi-outdoors or indoors) as the ground truth with timestamps. The IO detection error is introduced when the environmental type identified by the system is different from the ground truth. We defined the IO detection accuracy as the percentile when the system identifies the environmental type to be matched with the ground truth. The IO detection accuracy of BlueDetect is compared to GPS-IO [9] and IODetector [21]. The detection results of the three approaches are illustrated at the bottom of Figure 5 compared to the ground truth of the test walking route. It can be observed from Figure 5 that the IO detection accuracy of BlueDetect is the best among the three approaches. According to our experimental data, the overall IO detection accuracy of BlueDetect is 96.2%, which is much higher than GPS-IO (56.1%) and IODetector (78.8%). It is clear that BlueDetect can capture each transition between different environments precisely and effectively, be it from outdoors to semi-outdoors, semi-outdoors to indoors, indoors to semi-outdoors or semi-outdoors to outdoors.

In comparison, IODetector suffers from the misdetection of certain semi-outdoor and indoor contexts. The performance of GPS-IO is worst among the three approaches because the GPS signals are still high in some rooms with large windows; these indoor environments are easily misclassified as outdoor environments when the availability of GPS signals is the sole indicator for IO detection. Moreover, the performance of GPS-IO further degrades on rainy or cloudy days, because the LOS paths between the mobile device and satellites are blocked by thick cloud. It is noted that the influence of weather conditions on RSS of BLE beacons is negligible based on our experimental statistics.

### 5.2. Localization Accuracy of BlueDetect in Semi-Outdoor Environments

In addition to the IO detection accuracy analysis of BlueDetect, we also evaluate the localization accuracy of BlueDetect in semi-outdoor environments. Existing research works usually assume that GPS or WiFi-based IPS can deliver LBS in semi-outdoor environments [20,24,25]. However, this assumption hardly holds in reality. In semi-outdoor environments, the localization accuracy of GPS degrades severely, since some LOS paths to satellites are blocked by buildings, and WiFi-based IPS has a bottleneck due to the sparse deployment of WiFi APs in this scenario. Thus, the localization accuracy of sole GPS, WiFi or the integration of both is not precise and robust in semi-outdoor environments. On the contrary, by only employing a sparse density of BLE beacons, BlueDetect is able to provide accurate and reliable positioning and navigation service in semi-outdoor areas. We recorded the estimated locations of the mobile device by BlueDetect and GPS and compared these to the measured ground truth. The ground truth was obtained as follows: we first marked the ground with a 1-m grid along the test walking route and measured the physical coordinates of all grid points. Then, we used a camera to record the entire walking process. With the timestamps of grid points, the estimated locations of each grid point by BlueDetect and GPS are compared to its physical location. We define the location estimation error *e* to be the distance between the real location coordinates $\left(x_{0},y_{0}\right)$ and the system estimated location coordinates (x,y), *i.e.*, $e=\sqrt{{\left(x-x_{0}\right)}^{2}+{\left(y-y_{0}\right)}^{2}}$.

**Figure 8:**
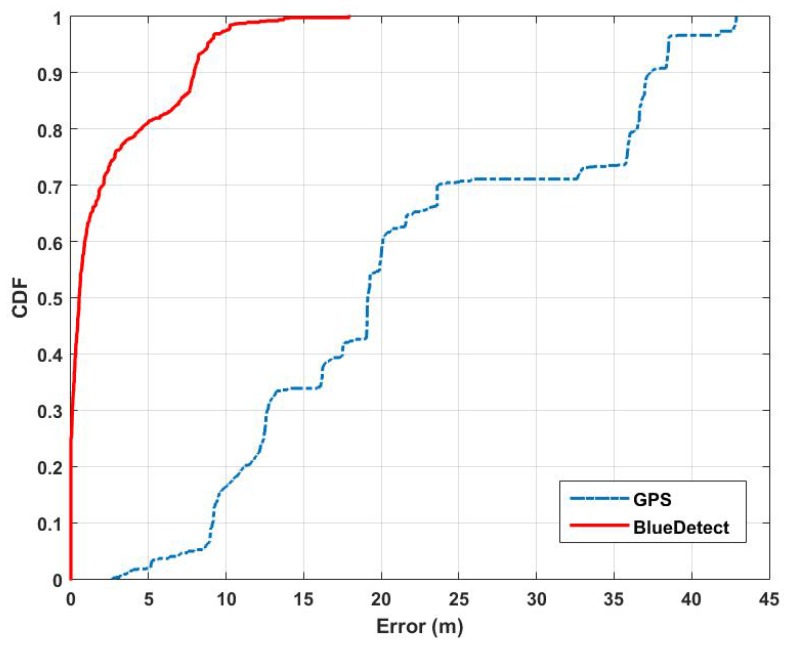
Comparison of the cumulative distribution of the location error between GPS and BlueDetect in semi-outdoor environments.

According to our experimental results, BlueDetect provides 2.18 m on average in semi-outdoor areas with an enhancement of accuracy around 89.87%, compared to GPS (21.53 m). Figure 8 depicts the cumulative distribution of the localization error of these two approaches. The distance error distribution of BlueDetect is mainly within 10 m with the 90th percentile of 7.94 m. On the contrary, the error distribution of GPS is much more scattered, where the 90th percentile is up to 37.33 m.

### 5.3. Power Consumption of BlueDetect

**Figure 9:**
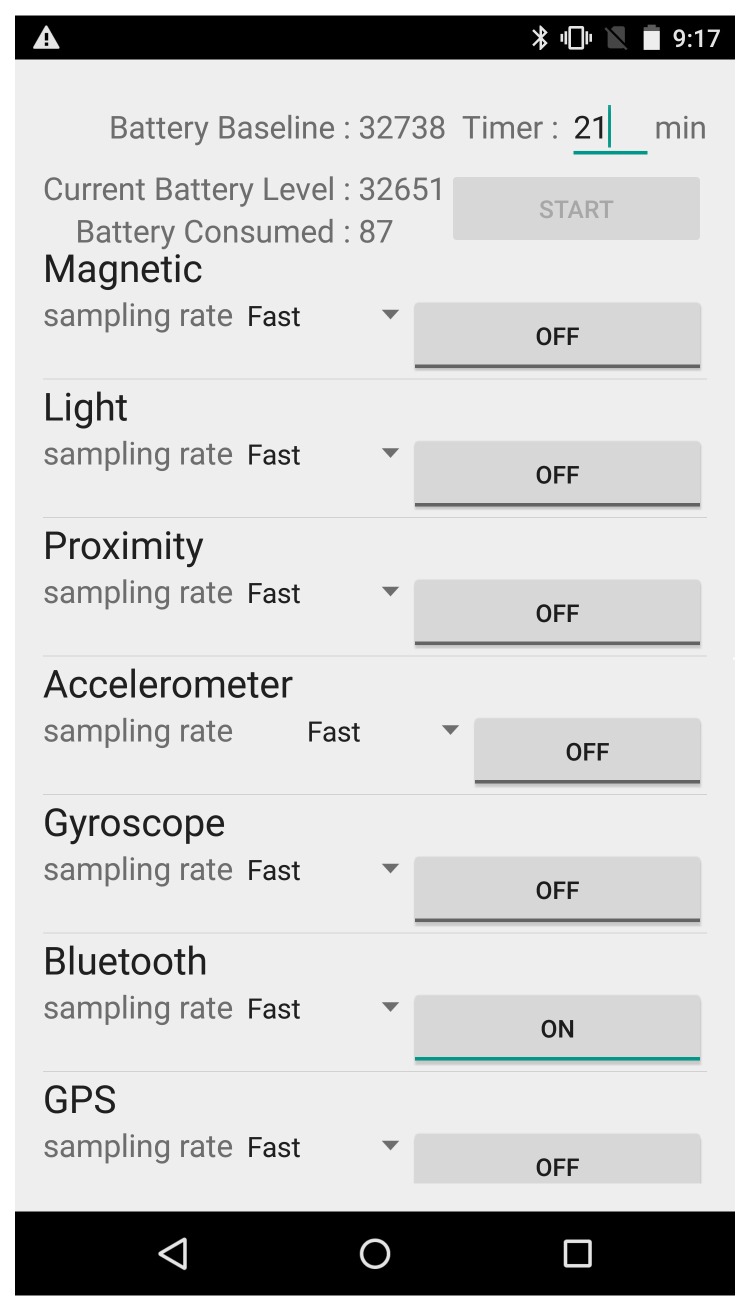
Screenshot of the power-monitoring app.

**Figure 10:**
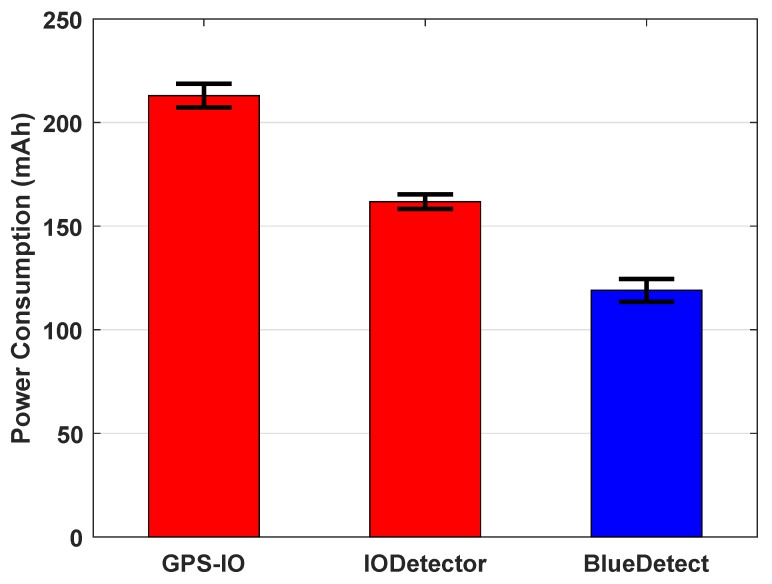
Power consumption of various sensors on a mobile device (Nexus 6) for different IO detection methods.

Nowadays, the battery consumption of an app is a critical metric to evaluate its feasibility. In this section, we analyze the energy consumption of BlueDetect and compared it to other approaches. BlueDetect performs IO detection by employing the Bluetooth module on mobile device; GPS-IO leverages the GPS module; and IODetector [21] utilizes the on-board light sensor and inertial measurement unit (IMU) sensors (including the accelerator, magnetometer and proximity sensors). In order to calculate how much power the mobile device needs for each approach, we develop a power-monitoring app that allows us to measure the battery consumption of certain on-board sensors precisely. The methodology is that after we select the sensors to be used for IO detection, the power-monitoring app records the battery readings when we start and finish the test walking route. The difference of these two readings is the power consumption of the IO detection scheme. The screen brightness is set at the minimum level during this process. This power consumption evaluation is conducted on a Nexus 6. Figure 9 is a screenshot of the power-monitoring app. The sensors adopted for each approach (GPS-IO, IODetector and BlueDetect) were turned on for 30 min and repeated five times. The average experimental data are adopted for evaluation. Figure 10 demonstrates how much power is consumed for the three IO detection methods.

As shown in Figure 10, the power consumption of BlueDetect is only 119 mAh in 30 min, which is the minimum among the three methods. On the contrary, the GPS module is the most power-hungry sensor, which consumed 213 mAh during one test walking route. GPS should be turned off in both semi-outdoor and indoor environments, since it provides useless information while drawing a huge amount of energy at the same time. The total energy consumption of IODetector is 162 mAh, which is distributed on light, cellular, magnetic field and accelerator and proximity sensors. Although its power consumption is less than GPS-IO, it consumes 26.54% more power than BlueDetect. Moreover, its detection accuracy is restricted by many factors. For instance, the light detector is useless if the mobile device is inside one’s pocket, while BlueDetect is immune to such a case.

In summary, the high detection accuracy and low energy consumption of BlueDetect makes it affordable for users and upper-layer applications.

## 6. Conclusion

In this paper, we propose BlueDetect as an accurate, fast response and energy-efficient scheme for IO detection and seamless LBS running on the mobile device based on the emerging low-power iBeacon technology. By leveraging the portable BLE beacons and Bluetooth module on mobile devices, BlueDetect provides precise IO detection results to turn on/off on-board sensors (such as WiFi and GPS) smartly, improve their performances and reduce the power consumption of mobile devices simultaneously. Furthermore, seamless LBS, such as positioning and navigation service, can be realized by BlueDetect, especially in semi-outdoor environments, which cannot be achieved by GPS or IPS easily. We prototyped BlueDetect on multiple Android mobile devices and analyzed its performance comprehensively. It provides higher IO detection accuracy, higher localization accuracy in semi-outdoor environments and consumes less battery than existing schemes. It is a feasible solution for IO detection and can be extended for other services, such as geo-fencing and floor identification, in the future.

## Figures and Tables

**Table 1:** Overview of technologies for location-based services (LBS).

Technology	Suitable Environment	Localization Accuracy	Extra Device on User-Side	Power Consumption	Cost
GPS	Outdoor	10 m	No	High	Moderate
GSM (cellular)	Outdoor and indoor	10–50 m	No	Low	Low
Infrared	Indoor	0.5–3 m	Yes	Low	Moderate
Acoustic signal	Indoor	30–80 cm	No	Low	Moderate
RFID	Indoor	1–3 m	Yes	Low	Moderate
UWB	Indoor	10–50 cm	Yes	Low	High
PDR	Indoor and outdoor	1–5 m	No	High	Low
WiFi	Indoor	2–5 m	No	High	Low
BLE (iBeacon)	Indoor and semi-outdoor	1–5 m	No	Low	Low

**Table 2:** Analysis of BLE beacon’s RSS variations under LOS and NLOS conditions.

**Distance from the Beacon**									
*(m)*	1	2	3	4	5	6	7	8	9
**Line-of-Sight (LOS)**									
*(dBm)*	−59	−62	−64	−66	−68	−70	−72	−75	−79
**None-Line-of-Sight (NLOS)**									
*(dBm)*	−63	−66	−68	−69	−72	−75	−77	−80	−83

**Table 3:** Analysis of BLE beacon’s RSS variations with the influence of different holding orientations of the mobile device.

Distance from the Beacon *(m)*	Orientation $0^{{}^{\circ}}$ *(dBm)*	Orientation $90^{{}^{\circ}}$ *(dBm)*	Orientation $180^{{}^{\circ}}$ *(dBm)*	Orientation $270^{{}^{\circ}}$ *(dBm)*
1	−59	−60	−63	−59
2	−62	−63	−66	−61
3	−64	−66	−68	−63
4	−66	−68	−69	−67
5	−68	−71	−72	−70
6	−70	−72	−75	−72
7	−72	−73	−77	−74
8	−75	−77	−80	−76
9	−79	−81	−83	−80
